# Systolic Ejection Murmurs Due to Unruptured Aneurysm of the Sinus of Valsalva

**DOI:** 10.7759/cureus.35796

**Published:** 2023-03-05

**Authors:** Masaki Noguchi, Tatsuya Kawasaki, Michiyo Yamano, Kazuki Maeda, Keisuke Kiyota

**Affiliations:** 1 Department of Cardiology, Matsushita Memorial Hospital, Moriguchi, JPN; 2 Central Clinical Laboratory, Matsushita Memorial Hospital, Moriguchi, JPN; 3 Department of Family Medicine, Kiyota Clinic, Moriguchi, JPN

**Keywords:** valsalva, symptom, physical examination, murmur, aneurysm

## Abstract

Aneurysm of the sinus of Valsalva is a rare condition with variable clinical presentation. We present a case of an unruptured aneurysm of the right sinus of Valsalva, in which a systolic ejection murmur was instrumental in the diagnosis. An asymptomatic 72-year-old man was referred to the cardiology department because of a heart murmur. Physical examination was unremarkable except for a grade 3 systolic murmur, loudest at the third left sternal border. Echocardiography revealed a sac-like structure protruding into the right ventricle and attached to the right sinus of Valsalva with a right ventricular outflow tract obstruction during end-systole. Multidetector computed tomography showed an aneurysm of the right sinus of Valsalva with a diameter of 28 × 19 mm; no contrast leakage from the aneurysm was detected. A diagnosis of an unruptured aneurysm of the right sinus of Valsalva was made. Surgical repair was successfully performed, and the murmur disappeared postoperatively. This case underscores the importance of physical examination even in the era of advanced imaging techniques and the need to recognize the wide range of causes of heart murmurs.

## Introduction

An aneurysm of the sinus of Valsalva, a condition characterized by the dilatation of the sinuses between the aortic valve annulus and the sinotubular junction, is a rare occurrence with varied clinical manifestations [1,2]. In this case report, we present a case of an unruptured aneurysm of the right sinus of Valsalva, in which systolic ejection murmurs were instrumental in the diagnosis.

## Case presentation

An asymptomatic 72-year-old man was referred to the cardiology department because he had been found to have heart murmurs. The patient denied any chest trauma. He had diabetes mellitus, hypertension, and a duodenal ulcer. Medications included amlodipine 10 mg daily, telmisartan 20 mg daily, and rabeprazole 10 mg daily. He quit smoking at the age of 50 years after a 45-pack-year history, drank occasionally, and had no known allergies. The patient had no family history of cardiovascular diseases.

The patient's body mass index was 17.8 kg/m^2^, and vital signs were within normal range. On physical examination, the jugular venous pressure was not elevated. A grade 3 systolic murmur was most audible at the third left sternal border; there were no additional heart sounds or rumbles audible. The auscultation of both lungs revealed clear breath sounds, and there was no edema in the lower extremities. Laboratory data were unremarkable except for a blood sugar level of 128 mg/dl and a glycated hemoglobin level of 7.1%. The brain natriuretic peptide level was 16.9 pg/ml (reference value: ≤18.4). Electrocardiogram and chest radiograph were normal.

**Figure 1:**
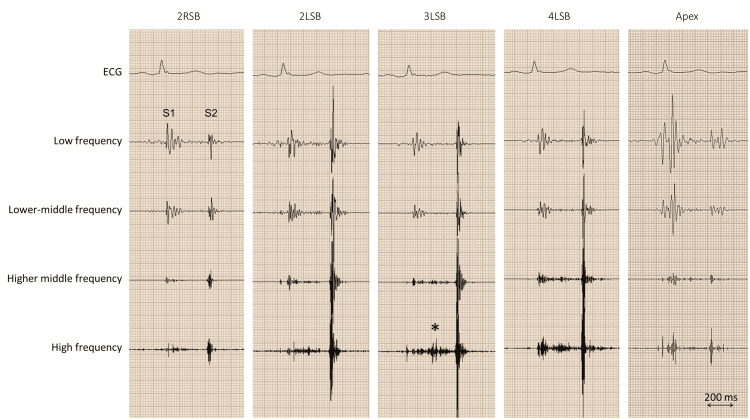
Phonocardiography A high-frequency, systolic ejection murmur (asterisk) is shown best on the third left sternal border (3LSB) 2LSB, second left sternal border; 2RSB, second right sternal border; 4LSB, fourth left sternal border; ECG, electrocardiogram; S1, first sound; S2, second sound

**Figure 2:**
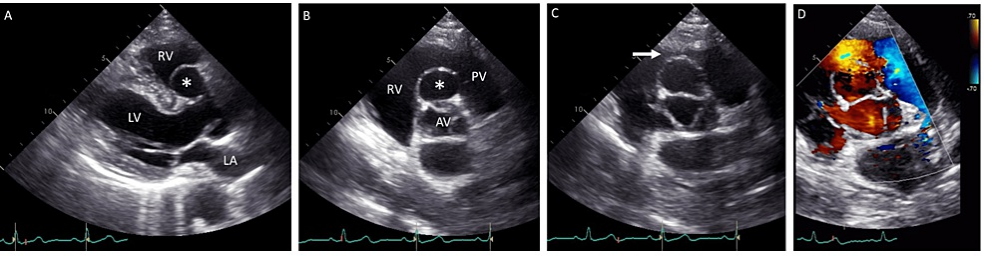
Echocardiography An echocardiographic examination reveals a sac-like structure in the right sinus of Valsalva on the parasternal long-axis view (A: asterisk) and its protrusion into the outflow tract of the right ventricle (RV) on the short-axis view at the aortic valve (AV) level (B: asterisk). Note the presence of obstruction at end-systole (C: arrow) and a mosaic pattern on color Doppler imaging (D) LA, left atrium; LV, left ventricle; PV, pulmonary valve

**Figure 3:**
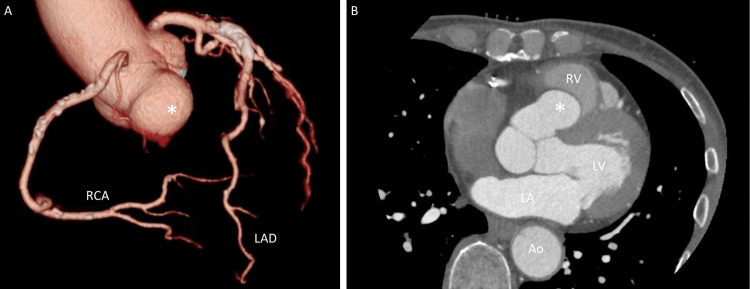
Computed tomography Three-dimensional reconstruction showed an aneurysm of the right sinus of Valsalva (A: asterisk). Note that there is no leakage of contrast medium from the aneurysm (B: asterisk) to the right ventricle (RV) on the axial image Ao, aorta; LA, left atrium; LAD, left anterior descending coronary artery; LV, left ventricle; RCA, right coronary artery

A phonocardiogram was performed to further evaluate the murmur, although not mandatory, and revealed a high-pitched systolic ejection murmur, loudest at the third left sternal border (Figure 1). An echocardiographic examination revealed normal ventricular function and size, with no evidence of valvular dysfunction or congenital disease. However, a sac-like structure protruding into the right ventricle and attached to the right sinus of Valsalva was observed (Figure 2A-2B). During end-systole, the obstruction of the right ventricular outflow tract was also noted (Figure 2C-2D). Multidetector computed tomography with three-dimensional reconstruction revealed an aneurysm of the right sinus of Valsalva with a diameter of 28 × 19 mm (Figure 3A); no leakage of contrast medium from the aneurysm was detected on axial images (Figure 3B). Thus, a diagnosis of an unruptured aneurysm of the right sinus of Valsalva was made.

Given the enlarged size of the sinus of Valsalva (i.e., 57 mm), surgical repair was performed at another hospital; the unruptured aneurysm was resected and replaced with an artificial vessel attached to the right coronary artery. The patient's clinical course was uneventful, and the murmurs disappeared after the operation. The patient has been doing well for over six months post surgery.

## Discussion

The current patient presented with a grade 3 systolic murmur best heard at the third left sternal border. While echocardiography was generally unremarkable, it did reveal a sac-like structure attached to the right sinus of Valsalva and protruding into the right ventricular outflow tract, causing obstruction but no shunt flow. Based on these findings, a diagnosis of an unruptured aneurysm of the right sinus of Valsalva was made. The location of the murmurs and their resolution after surgical repair suggest that the heart murmur was likely attributed to the unruptured aneurysm of the right sinus of Valsalva.

Symptoms associated with a Valsalva aneurysm may range from asymptomatic status to cardiogenic shock, and this variability may be due to clinically distinct conditions among patients with a Valsalva aneurysm, such as ruptured versus unruptured aneurysm or large versus small shunt. To investigate the initial symptoms of this condition, a systematic literature search was conducted on PubMed using the terms "Valsalva aneurysm" and "case report." A total of 103 reports were identified, of which 61 were excluded due to a ruptured aneurysm of the sinus of Valsalva in 50 reports, another diagnosis in two reports, an animal report in one, and a lack of patient information in eight reports.

The 42 included reports comprised 46 patients with an unruptured aneurysm of the sinus of Valsalva [3-44]. Among the patients with an unruptured aneurysm, the most common symptom or condition leading to diagnosis was chest pain in 15, dyspnea in 12, follow-up for another cardiac disease (e.g., Marfan syndrome) in six, atrioventricular block in five, heart murmurs in two, and dizziness or fainting in two. Interestingly, the presence of a heart murmur may have led to the diagnosis of an unruptured Valsalva aneurysm in two patients.

It is well known that loud, typically continuous, heart murmurs can occur after the rupture of an aneurysm. However, it is unclear whether a new onset of heart murmurs could be a diagnostic sign in patients with an unruptured Valsalva aneurysm. It should be noted that in these two patients in whom heart murmurs may have led to the diagnosis of an unruptured Valsalva aneurysm, the murmurs were not related to the aneurysm but to other concomitant conditions (i.e., pulmonary valve endocarditis [32] and aortic regurgitation and stenosis [39]). To the best of our knowledge, this case represents the first report demonstrating that an unruptured Valsalva aneurysm can be detected through the physical examination of this rare entity. Although phonocardiography provided the detailed characteristics of the murmur in this case, it is worth noting that this analysis was not mandatory for the diagnostic process due to the low availability of phonocardiography.

## Conclusions

The present case underscores the importance of physical examination in modern times, even in the presence of advanced imaging techniques, as clinical practice without physical examination may result in incorrect or delayed diagnoses.
